# A dual functional redox enzyme maturation protein for respiratory and assimilatory nitrate reductases in bacteria

**DOI:** 10.1111/mmi.14239

**Published:** 2019-04-06

**Authors:** Benjamin J. Pinchbeck, Manuel J. Soriano‐Laguna, Matthew J. Sullivan, Victor M. Luque‐Almagro, Gary Rowley, Stuart J. Ferguson, M. Dolores Roldán, David J. Richardson, Andrew J. Gates

**Affiliations:** ^1^ Centre for Molecular and Structural Biochemistry University of East Anglia Norwich Research Park Norwich NR4 7TJ UK; ^2^ School of Biological Sciences University of East Anglia Norwich Research Park Norwich NR4 7TJ UK; ^3^ School of Medical Science Gold Coast campus, Griffith University Southport, QLD 4222 Australia; ^4^ Departamento de Bioquímica y Biología Molecular, Edificio Severo Ochoa, 1 planta, Campus de Rabanales Universidad de Córdoba Córdoba 14071 Spain; ^5^ Department of Biochemistry University of Oxford South Parks Road Oxford OX1 3QU UK

## Abstract

Nitrate is available to microbes in many environments due to sustained use of inorganic fertilizers on agricultural soils and many bacterial and archaeal lineages have the capacity to express respiratory (Nar) and assimilatory (Nas) nitrate reductases to utilize this abundant respiratory substrate and nutrient for growth. Here, we show that in the denitrifying bacterium *Paracoccus denitrificans*, NarJ serves as a chaperone for both the anaerobic respiratory nitrate reductase (NarG) and the assimilatory nitrate reductase (NasC), the latter of which is active during both aerobic and anaerobic nitrate assimilation. Bioinformatic analysis suggests that the potential for this previously unrecognized role for NarJ in functional maturation of other cytoplasmic molybdenum‐dependent nitrate reductases may be phylogenetically widespread as many bacteria contain both Nar and Nas systems.

## Introduction

**Figure 1:**
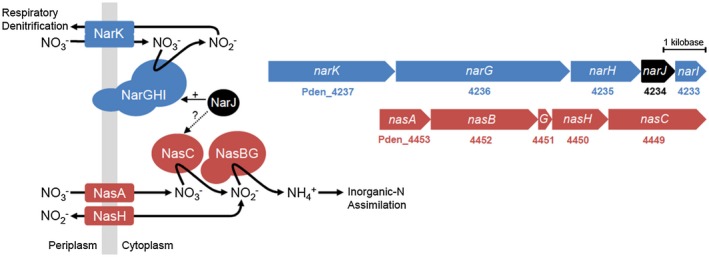
Genetic and biochemical organization of the structural components for the *P. denitrificans* Nar and Nas systems.

The denitrifying α‐proteobacterium *Paracoccus denitrificans* Pd1222 contains two distinct nitrate (${\text{NO}}_{3}^{-}$) reductases that have active sites in the cytoplasmic cellular compartment (Richardson *et al.*, 2001). A membrane‐bound ${\text{NO}}_{3}^{-}$ reductase system (Nar) functions in anaerobic respiration and an assimilatory ${\text{NO}}_{3}^{-}$ reductase system (Nas) is involved in the incorporation of inorganic nitrogen (N) into biomass (Gates *et al.*, 2011). Both redox proteins catalyze the two‐electron reduction of ${\text{NO}}_{3}^{-}$ to nitrite (${\text{NO}}_{2}^{-}$), but Nar draws the electrons required for ${\text{NO}}_{3}^{-}$ reduction from ubiquinol (Craske and Ferguson, 1986), while Nas acquires electrons from the reduced nicotinamide adenine dinucleotide (NADH) pool (Gates *et al.*, 2011). Although the two systems are different in terms of their biochemical properties, in both cases the catalytic subunit for ${\text{NO}}_{3}^{-}$ reduction, i.e. NarG for respiration and NasC for assimilation, is a member of the pyranopterin guanosine dinucleotide, or PGD, cofactor‐binding protein superfamily (Grimaldi *et al.*, 2013). Importantly, the respiratory ubiquinol/${\text{NO}}_{3}^{-}$ oxidoreductase NarGHI, encoded as part of the *narKGHJI* operon, is similar to that present in *Escherichia coli* (Craske and Ferguson, 1986), and requires additional proteins including NarK and NarJ for cellular function. Here, NarK and NarJ are tasked with ${\text{NO}}_{3}^{-}$/${\text{NO}}_{2}^{-}$ transport across the cytoplasmic membrane and assembly of the active metalloenzyme complex respectively, Fig. 1 (Berks *et al.*, 1995). Under anaerobic conditions in *Paracoccus* sp., ${\text{NO}}_{3}^{-}$ reduction by Nar is coupled to generation of a trans‐membrane electrochemical potential and is the first‐step in the denitrification pathway (Berks *et al.*, 1995). The ${\text{NO}}_{2}^{-}$ generated is exported to the periplasm by NarK (Wood *et al.*, 2002), where it is further reduced to dinitrogen gas via the intermediates nitric oxide and nitrous oxide by the concerted action of the respiratory nitrite (NirS), nitric oxide (NorCB) and nitrous oxide (NosZ) reductases, respectively (Berks *et al.*, 1995).

By contrast, NasC, the catalytic subunit of the assimilatory NADH‐dependent ${\text{NO}}_{3}^{-}$ reductase is encoded by the *nasABGHC* operon, which also codes for a ${\text{NO}}_{3}^{-}$/${\text{NO}}_{2}^{-}$ transporter (NasA), a ${\text{NO}}_{2}^{-}$ transporter (NasH), a ${\text{NO}}_{2}^{-}$ reductase (NasB) and an electron‐transfer ferredoxin (NasG) (Fig. 1). The ${\text{NO}}_{2}^{-}$ generated from ${\text{NO}}_{3}^{-}$ reduction by NasC remains in the cytoplasm and is further reduced by NasB to ammonium (${\text{NH}}_{4}^{+}$), which is incorporated into biomass via transamination reactions (Gates *et al.*, 2011). NasC and NasB both receive electrons from the NADH pool for their respective reactions and may form a complex together with the electron‐transfer ferredoxin NasG (Gates *et al.*, 2011). Despite the differences between the Nar and Nas systems, biochemical analyses have suggested that there can be functional ‘cross‐talk’ between the two systems *in vivo*, in terms of ${\text{NO}}_{3}^{-}$ uptake and its reduction to ${\text{NO}}_{2}^{-}$, when they are co‐expressed under anaerobic growth conditions with ${\text{NO}}_{3}^{-}$ serving as both respiratory electron acceptor and sole N‐source for growth (Gates *et al.*, 2011). In addition, in many heterotrophic bacteria, additional genes are present within the *nas* gene cluster, e.g. *cysG* that are predicted to encode proteins for biosynthesis of the siroheme cofactor required for functional maturation of the assimilatory ${\text{NO}}_{2}^{-}$ reductase holoenzyme (Luque‐Almagro *et al.*, 2011).

Correct folding of complex redox proteins and introduction of cofactors can require chaperones called Redox Enzyme Maturation Proteins (REMPs) (Bay *et al.*, 2015). NarG is a well‐studied molybdenum (Mo) *bis*‐PGD (Mo[PGD]_2_) binding protein synthesized during anaerobiosis in a range of microorganisms. In comparison, NasC is relatively understudied but the closely‐related cyanobacterial protein NarB is known to bind a Mo[PGD]_2_ active site cofactor (Jepson *et al.*, 2004). In addition, both respiratory and assimilatory ${\text{NO}}_{3}^{-}$ reductases bind one or more iron‐sulfur cluster(s) to facilitate electron transfer to the active site. For the respiratory ${\text{NO}}_{3}^{-}$ reductase NarG, it has been previously demonstrated that insertion of the Mo[PGD]_2_ cofactor is performed by the chaperone NarJ in *E. coli* (Blasco *et al.*, 1992; Blasco *et al.*, 1998; Bay *et al.*, 2015). In *P. denitrificans*, *narJ* (Pden_4234) also forms an integral part of the *narKGHJI* gene cluster. However, there is no gene present within the assimilatory *nasABGHC* gene cluster predicted to encode a REMP for NasC, and no other obvious candidates can be found elsewhere in the genome. This study aims to explore the possibility that in *P. denitrificans*, NarJ is a uniquely dual‐functional REMP, which can chaperone maturation of both assimilative and dissimilative ${\text{NO}}_{3}^{-}$ reductases that are catalytically active in the cytoplasm.

## Results

### The Mo‐dependence of assimilatory and respiratory nitrate reduction in *P. denitrificans*

**Figure 2:**
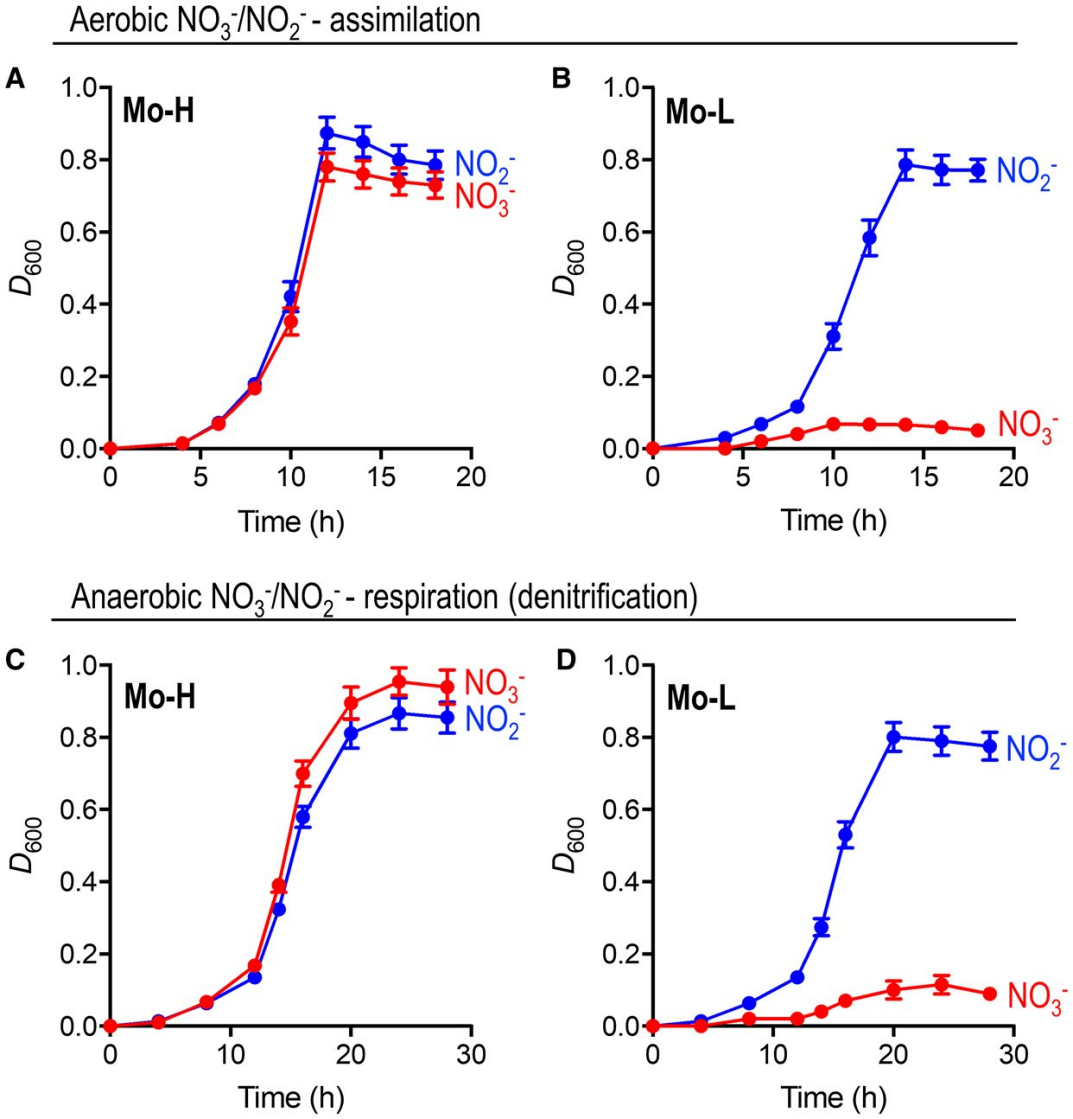
Bacterial growth in response to Mo availability. *P. denitrificans* was grown at 30°C in batch culture containing minimal salt media supplemented with 30 mM of succinate as carbon source and either Mo‐H (A and C) or Mo‐L (B and D) trace‐metal solutions. Growth curves are presented for aerobic assimilation (A and B) with 10 mM of ${\text{NO}}_{3}^{-}$ (red) or ${\text{NO}}_{2}^{-}$ (blue) as sole N‐sources. Growth was also measured under anaerobic denitrifying conditions (C and D) using 10 mM of ${\text{NH}}_{4}^{+}$ as N‐source and 20 mM of ${\text{NO}}_{3}^{-}$ (red) or ${\text{NO}}_{2}^{-}$ (blue) as respiratory electron acceptor.

NasC is a putative Mo[PGD]_2_ binding protein expressed under aerobic ${\text{NO}}_{3}^{-}$‐dependent growth conditions required for biomass production, while NarG is a well‐studied Mo[PGD]_2_ binding protein synthesized during anaerobiosis to couple the reduction of ${\text{NO}}_{3}^{-}$ with cellular respiration. To explore the metabolic importance of Mo to *P. denitrificans* under different N growth regimes, comparative experiments were performed using minimal salts media prepared with or without inclusion of the trace metal salt sodium molybdate. Figure 2 shows growth curves for *P. denitrificans *cultured under ${\text{NO}}_{3}^{-}$/${\text{NO}}_{2}^{-}$‐dependent assimilatory and denitrifying conditions. Bacterial growth was readily observed during aerobic or anaerobic conditions with ${\text{NO}}_{3}^{-}$ as assimilatory or respiratory substrate in Mo‐H minimal media (Fig. 2A and C). However, the absence of Mo (in Mo‐L cultures) abolished growth by both metabolic modes (Fig. 2B and D). Where ${\text{NO}}_{2}^{-}$ was used as N‐source and/or respiratory electron acceptor, growth was similar and significant regardless of the Mo‐content of media (Fig. 2). As aerobic and anaerobic growth with ${\text{NO}}_{2}^{-}$ was unaffected, these observations suggest that the assimilatory and respiratory ${\text{NO}}_{3}^{-}$ reductase systems are Mo‐dependent in *P. denitrificans *and in the absence of a dedicated REMP for NasC would perhaps point toward a common chaperone to drive incorporation of the Mo[MGD]_2_ cofactor into both enzymes.

### Expression of *narJ* during nitrate assimilation

**Figure 3:**
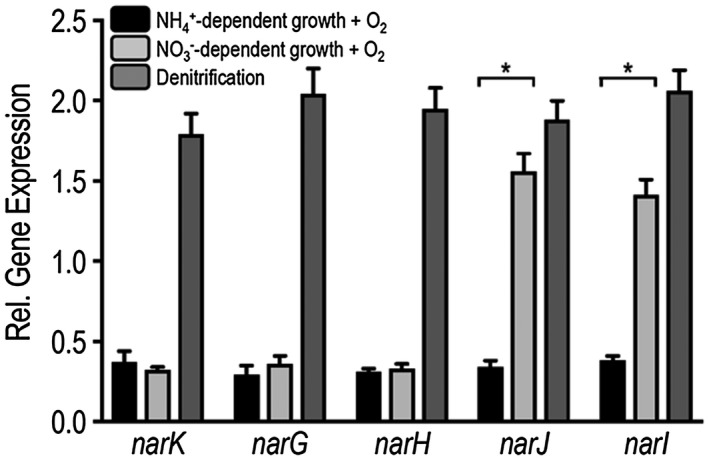
Comparative analysis of *nar* gene expression by qRT‐PCR during ${\text{NO}}_{3}^{-}$ assimilation and respiration. (A) *P. denitrificans* was grown in batch culture containing minimal salt media with 30 mM of succinate at 30°C. For ${\text{NO}}_{3}^{-}$ assimilation, cells were grown aerobically with either 10 mM of ${\text{NH}}_{4}^{+}$ (control condition, black) or ${\text{NO}}_{3}^{-}$ (light grey) as sole N‐sources. For denitrification, both ${\text{NH}}_{4}^{+}$ and ${\text{NO}}_{3}^{-}$ were included during anaerobic cell culture (dark grey). RNA was prepared from cells harvested at mid‐exponential phase and *nar* gene expression was normalized relative to *dnaN*, a housekeeping gene. Data presented are the average of three biological replicates, where ^*^ *P* ≤ 0.005.

The *P. denitrificans narKGHJI* gene cluster is known to be expressed under anaerobic conditions during ${\text{NO}}_{3}^{-}$ respiration and is regulated by the oxygen and ${\text{NO}}_{3}^{-}$/${\text{NO}}_{2}^{-}$ responsive transcription factors FnrP and NarR respectively (Wood *et al.*, 2001), consistent with the key role of these genes in denitrification (Giannopoulos *et al.*, 2017). By contrast, the *nasABGHC* genes are expressed in response to the C/N‐status of the cell and independently to oxygen tension when ${\text{NO}}_{3}^{-}$ or ${\text{NO}}_{2}^{-}$ is present (Gates *et al.*, 2011; Luque‐Almagro *et al.*, 2013). Thus, in order for NarJ to potentially chaperone the incorporation of Mo[MGD]_2_ during aerobic synthesis of NasC, *narJ* expression would have to overcome aerobic repression of the *narK* promotor. Expression analysis of the *narKGHJI* genes was therefore undertaken under a range of growth conditions. As expected, qRT‐PCR data indicated that when *P. denitrificans* is grown aerobically with ${\text{NH}}_{4}^{+}$ as sole N‐source, only a low level of expression of the *nar* gene cluster is observed (Fig. 3, black). Expression of all genes increased around 4 fold, under standard denitrifying conditions, i.e. during anaerobic growth with ${\text{NH}}_{4}^{+}$ as N‐source and ${\text{NO}}_{3}^{-}$ present as respiratory electron acceptor (Fig. 3, light grey). When grown aerobically with ${\text{NO}}_{3}^{-}$ as sole N‐source and where functional NasC is required for growth, *narKGH* exhibited similar low basal level expression to that observed when ${\text{NH}}_{4}^{+}$ was the sole N‐source in control experiments. Notably, though, expression of both *narJ* and *narI* was significantly upregulated by ~4‐fold, comparable to levels observed under anaerobic ${\text{NO}}_{3}^{-}$ respiration (Fig. 3, dark grey). This demonstrates expression of *narJ* during aerobic ${\text{NO}}_{3}^{-}$‐dependent growth, i.e. under Nas‐dependent growth conditions. Gene expression of *narJI* is likely driven from an oxygen‐insensitive promotor downstream of the anaerobically active *narK* promotor, and points to a role for NarJ (and conceivably NarI) during both anaerobic and aerobic metabolism.

### Comparative growth of wild‐type and ∆*narJ* strains during nitrate assimilation and denitrifying conditions

**Figure 4:**
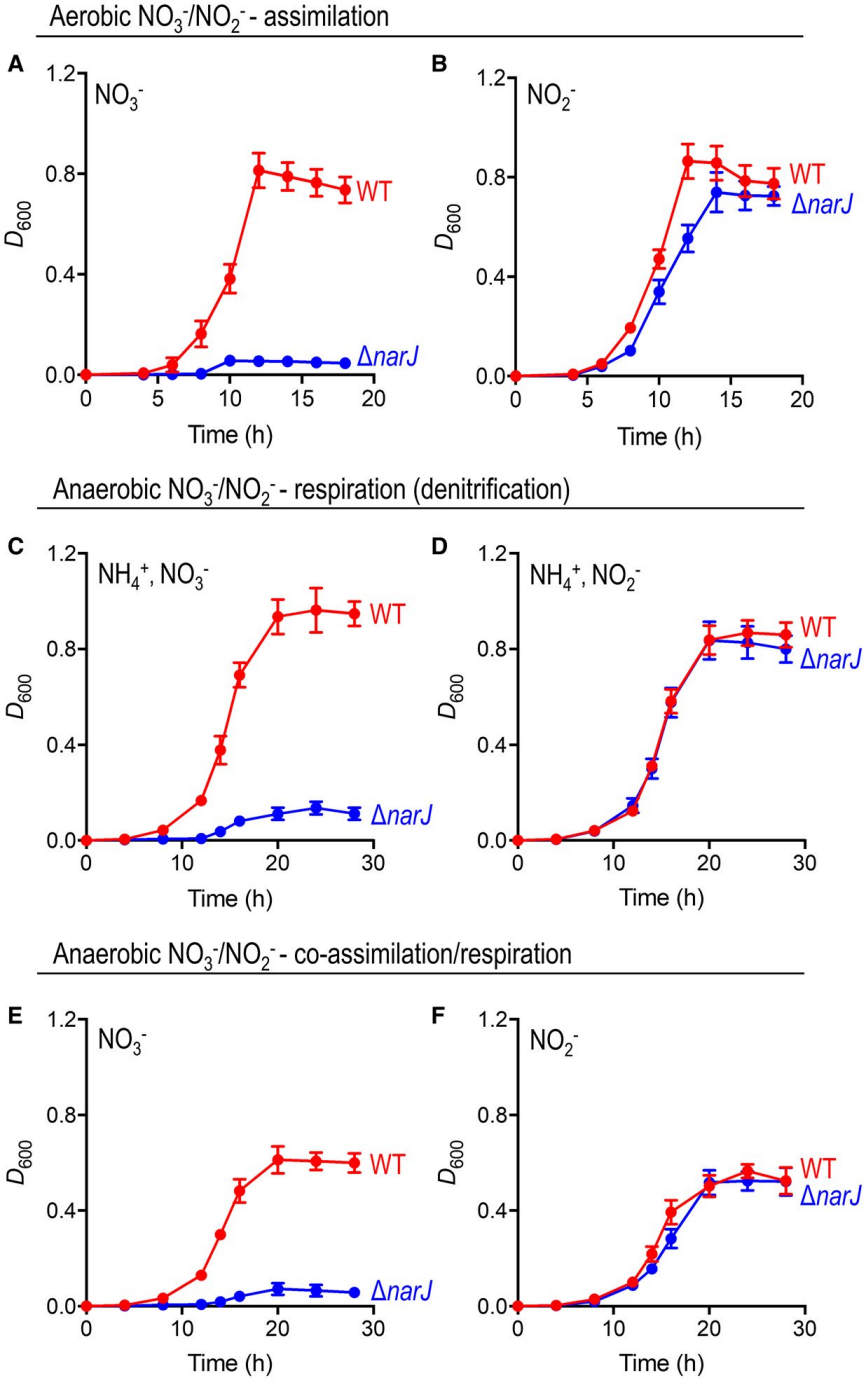
Comparative growth curves for WT and ∆*narJ* strains performing assimilation, respiration and co‐assimilation/respiration of ${\text{NO}}_{3}^{-}$ or ${\text{NO}}_{2}^{-}$. WT (red) and ∆*narJ* (blue) strains were grown in batch culture with minimal salts medium containing 30 mM of succinate at 30°C. For ${\text{NO}}_{3}^{-}$/${\text{NO}}_{2}^{-}$ assimilation, cells were grown aerobically with 10 mM of ${\text{NO}}_{3}^{-}$ (A) or ${\text{NO}}_{2}^{-}$ (B) as sole N‐source. For ${\text{NO}}_{3}^{-}$/${\text{NO}}_{2}^{-}$ respiration (denitrification), cells were grown anaerobically on 20 mM of ${\text{NO}}_{3}^{-}$ (C) or ${\text{NO}}_{2}^{-}$ (D) with 10 mM of ${\text{NH}}_{4}^{+}$ also present to prevent ${\text{NO}}_{3}^{-}$/${\text{NO}}_{2}^{-}$ assimilation. For ${\text{NO}}_{3}^{-}$/${\text{NO}}_{2}^{-}$ co‐assimilation/respiration, cells were grown anaerobically with 30 mM of ${\text{NO}}_{3}^{-}$ (E) or ${\text{NO}}_{2}^{-}$ (F) as sole N‐source and respiratory electron acceptor. Results shown are the average of three biological replicates.

An unmarked, non‐polar *narJ* deletion mutant was constructed and comparative growth versus wild‐type (WT) was tested under aerobic and anaerobic conditions with either ${\text{NO}}_{3}^{-}$ or ${\text{NO}}_{2}^{-}$ present in culture media (Fig. 4). Firstly, the capacity of the ∆*narJ* strain to grow aerobically and assimilate N using ${\text{NO}}_{3}^{-}$ or ${\text{NO}}_{2}^{-}$ as sole N‐source was assessed. Under aerobic conditions, this strain clearly lost the ability to grow using ${\text{NO}}_{3}^{-}$ as sole N‐source (Fig. 4A). However, aerobic growth with ${\text{NO}}_{2}^{-}$ as sole N‐source was not impaired (Fig. 4B), and growth curves were essentially identical to those obtained for Nas‐independent growth of *P. denitrificans* with ${\text{NH}}_{4}^{+}$ (data not shown). Similarly, under denitrifying conditions, the ∆*narJ* strain was unable to grow anaerobically using ${\text{NO}}_{3}^{-}$ as respiratory electron acceptor (Fig. 4C), but it retained the ability to respire ${\text{NO}}_{2}^{-}$ (Fig. 4D). This is consistent with abolition of Nar‐dependent growth, and deliberate inclusion of millimolar levels of ${\text{NH}}_{4}^{+}$ in these experiments was used to supress Nas expression (Gates *et al.*, 2011). In the absence of ${\text{NH}}_{4}^{+}$, the ability of WT cells to co‐assimilate/respire ${\text{NO}}_{3}^{-}$ was also lost in the ∆*narJ* strain (Fig. 4E). The capacity for cells to co‐assimilate/respire ${\text{NO}}_{2}^{-}$ was unaffected (Fig. 4F). Notably, a ~ 40% drop in maximum growth yield was observed for strains performing co‐assimilation/respiration of ${\text{NO}}_{3}^{-}$ or ${\text{NO}}_{2}^{-}$ versus growth solely by ${\text{NO}}_{3}^{-}$ or ${\text{NO}}_{2}^{-}$ respiration (i.e. compare Fig. 4C with 4E for WT; and Fig. 4D with F for WT or ∆*narJ*). This observation is consistent with competition for substrate between Nas/Nar, and Nas/Nir, during co‐assimilation/respiration of ${\text{NO}}_{3}^{-}$ and ${\text{NO}}_{2}^{-}$ respectively.

**Figure 5:**
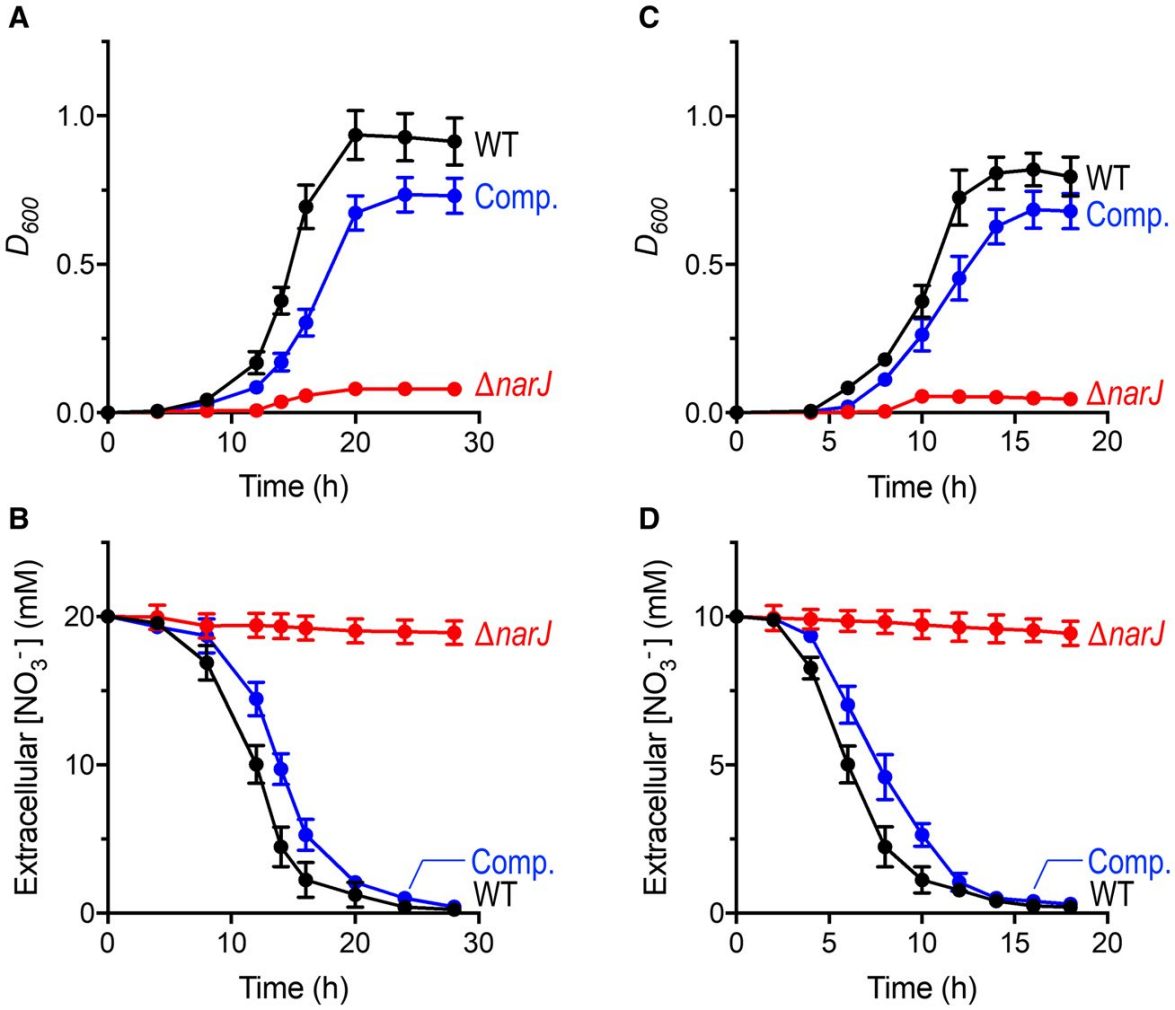
Comparative growth curves and ${\text{NO}}_{3}^{-}$‐uptake profiles for WT, ∆*narJ*, and complemented ∆*narJ*/pLMB509‐*narJ* strains. WT (black), ∆*narJ* (red) and ∆*narJ*/pLMB509‐*narJ* (Comp., blue) strains were grown in batch culture with minimal salts medium containing 30 mM of succinate at 30°C. For ${\text{NO}}_{3}^{-}$ respiration, cells were grown anaerobically with 20 mM of ${\text{NO}}_{3}^{-}$ as electron acceptor and 10 mM of ${\text{NH}}_{4}^{+}$ as N‐source (A) and corresponding media concentrations of ${\text{NO}}_{3}^{-}$ monitored (B). For ${\text{NO}}_{3}^{-}$ assimilation, cells were grown aerobically with 10 mM of ${\text{NO}}_{3}^{-}$ as sole N‐source (C) and media concentrations of ${\text{NO}}_{3}^{-}$ also monitored (D). All cultures contained an additional supplement of 1 mM taurine to stimulate *narJ* expression from the inducible complementation plasmid in the ∆*narJ*/pLMB509‐*narJ* strain. Results shown are the average of three biological replicates.

When the *narJ* gene was re‐introduced into the ∆*narJ* strain *in trans*, the ability of cells to preform both assimilatory ${\text{NO}}_{3}^{-}$ reduction and ${\text{NO}}_{3}^{-}$ respiration, under Nas‐dependent and Nar‐dependent growth conditions was recovered (Fig. 5). In both cases, growth curves (Fig. 5A and C) and growth‐linked ${\text{NO}}_{3}^{-}$‐uptake profiles (Fig. 5B and D) for the complemented ∆*narJ*/pLMB509‐*narJ *strain (Comp.) showed very similar characteristics to WT. Collectively, this data therefore reaffirms the role of NarJ in respiratory ${\text{NO}}_{3}^{-}$ reduction, but also presents a clear role for this chaperone in Nas‐dependent growth at the level of assimilatory ${\text{NO}}_{3}^{-}$ reduction.

### The impact of *narJ* deletion on assimilatory and respiratory nitrate reductase activity

**Figure 6:**
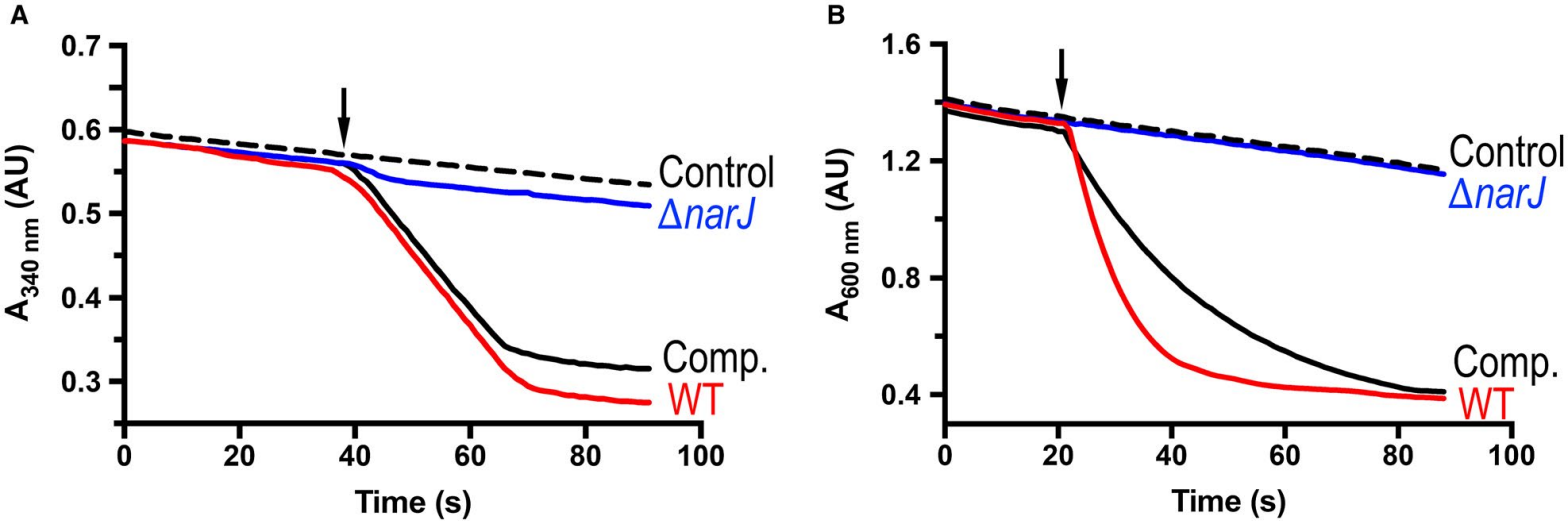
Determination of ${\text{NO}}_{3}^{-}$ reductase activity in lysates from cells performing ${\text{NO}}_{3}^{-}$ assimilation or respiration. Representative traces for NADH or reduced MV‐dependent ${\text{NO}}_{3}^{-}$ reductase activity present in WT (red), ∆*narJ* (blue), and ∆*narJ*/pLMB509‐*narJ* (black, solid) lysates, where cells were grown under aerobic ${\text{NO}}_{3}^{-}$ assimilation (A) or anaerobic ${\text{NO}}_{3}^{-}$ respiring conditions (B). Assays were initiated by addition of 1 mM ${\text{NO}}_{3}^{-}$ (denoted by the arrow) or buffer was injected in control assays (black, dashed). NADH or reduced MV dependent ${\text{NO}}_{3}^{-}$ reduction was followed at 340 or 600 nm, respectively. Measurements were performed in 20 mM HEPES, 150 mM NaCl (pH 7.5) buffer in anaerobic reactions at 20°C.

To directly assess the levels of active ${\text{NO}}_{3}^{-}$ reductase synthesized in WT, ∆*narJ* and ∆*narJ*/pLMB509‐*narJ *strains, a series of spectrophotometric solution assays with either reduced MV or NADH (for NasC and NasB) as electron donor were performed on whole cell lysates (Fig. 6). To investigate Nas and Nar activity, all strains were grown under aerobic and anaerobic conditions with ${\text{NO}}_{2}^{-}$ respectively. Notably, these conditions support growth of the ∆*narJ* strain to provide biomass for preparation of cell lysates. For growth of anaerobic denitrifying cells, 10 mM ${\text{NH}}_{4}^{+}$ was included to suppress Nas expression. A taurine supplement was also used to induce expression of *narJ* in the complemented ∆*narJ*/pLMB509‐*narJ *strain.

WT cells showed NADH‐dependent ${\text{NO}}_{3}^{-}$ (15.4 ± 1.2 nmol.min^−1^.mg protein^−1^) and ${\text{NO}}_{2}^{-}$ (54.1 ± 2.6 nmol.min^−1^.mg protein^−1^) reductase activities that were comparable to *P. denitrificans* cells grown aerobically with ${\text{NO}}_{3}^{-}$ as sole N‐source in previous studies (Gates *et al.*, 2011). However, in contrast to WT cells, extracts prepared from the ∆*narJ* strain showed no detectable NADH‐dependent ${\text{NO}}_{3}^{-}$ reductase activity (Fig. 6A), indicating that NasC was inactive. Similarly, MV‐dependent assays using denitrifying cell lysates also showed no detectable ${\text{NO}}_{3}^{-}$ reductase activity (Fig. 6B), consistent with both NasC and NarG being inactive in the absence of NarJ. Importantly, both NADH‐dependent and MV‐dependent ${\text{NO}}_{3}^{-}$ reductase activities for the ∆*narJ*/pLMB509‐*narJ *strain were similar to levels observed for WT cells grown under equivalent conditions. In addition, the levels of NADH‐dependent or MV‐dependent ${\text{NO}}_{2}^{-}$ reductase activity present in ${\text{NO}}_{2}^{-}$ assimilating (or denitrifying) cell lysates were very similar for all strains and consistent with previous studies performed on soluble extracts from *P. denitrificans* (Gates *et al.*, 2011).

### Molecular interaction of NarJ with NarG and NasC

**Figure 7:**
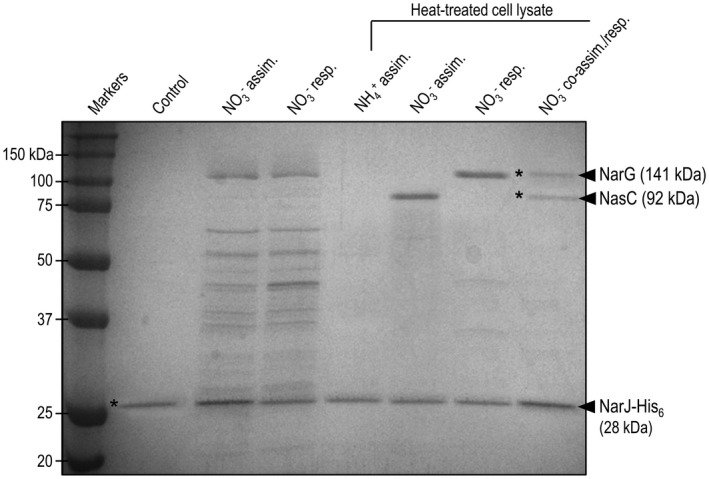
SDS‐PAGE analysis of NarJ interactions with assimilatory and respiratory ${\text{NO}}_{3}^{-}$ reductases in pull‐down assays. Magnetic beads were charged with ~5 μM of purified NarJ‐His_6_, washed and incubated with cell lysate prepared from *P. denitrificans *WT grown under either aerobic ${\text{NO}}_{3}^{-}$ assimilation (lanes 3 and 6), anaerobic ${\text{NO}}_{3}^{-}$ respiration (lanes 4 and 7) and anaerobic ${\text{NO}}_{3}^{-}$ co‐respiration/assimilation (lane 8) culture conditions. Control incubations were performed with buffer (lane 2) or lysate from aerobically grown cells assimilating ${\text{NH}}_{4}^{+}$ (lane 5). Samples where lysate underwent heat‐treatment prior to incubation with NarJ‐loaded beads are highlighted within the figure. The NarJ‐loaded beads were washed with buffer containing 0.5 M imidazole to elute NarJ and specific partner proteins for analysis by SDS‐PAGE. Precision Plus^TM^ dual color standards were loaded into lane 1. Asterisks denote bands excised for identification by mass spectrometry.

The metabolic phenotypes and enzymatic properties of the ∆*narJ* strain suggested a key role for NarJ in the assembly of active NarG and NasC. To explore whether functional maturation of these ${\text{NO}}_{3}^{-}$ reductases may involve a direct molecular interaction with NarJ, a recombinant NarJ‐His_6_‐tagged protein was synthesized and purified to homogeneity. Protein‐protein interaction studies were then performed using the NarJ‐His_6_ variant immobilized onto magnetic IMAC‐sepharose beads that were pre‐charged with Ni^2+^. This NarJ‐loaded support was then incubated with lysates from *P. denitrificans* WT cells cultured under either ${\text{NO}}_{3}^{-}$‐dependent assimilation, respiration or co‐assimilation/respiration growth conditions to stimulate appropriate NasC, NarG or NasC and NarG expression, respectively. In addition, selected lysate samples were briefly heat‐treated prior to mixing with NarJ‐loaded beads, to enable recognition of specific secondary epitopes in the partially unfolded state of potential target protein(s). Following incubation with lysates and subsequent wash steps, NarJ was released from the bead support, using imidazole, and bound proteins were visualized by SDS‐PAGE analysis (Fig. 7). Control experiments in which the NarJ‐support was incubated with buffer, rather than cell lysate, confirmed that a 25 kDa band was released from the beads following treatment with imidazole (Fig. 7, lane 2). This band was confirmed to be NarJ by MALDI‐TOF mass spectrometry (where a MASCOT significance score of 183, 67% coverage and expect value of 6.2 × 10^−10^ was obtained). Regardless of cell growth conditions, the use of non‐heat‐treated lysates resulted in weak non‐specific binding to the NarJ‐loaded support (e.g. see Fig. 7, lanes 3 and 4). However, a ~ 92 kDa band was reproducibly retained by NarJ‐loaded beads when incubated with heat‐treated lysate prepared form cells grown under aerobic ${\text{NO}}_{3}^{-}$ assimilation conditions (Fig. 7, lane 6), which was absent in lysates form cells grown under aerobic ${\text{NH}}_{4}^{+}$‐dependent growth conditions where expression of the *nas *gene cluster is repressed (Fig. 7, lane 5). This band was excised and analyzed by mass spectrometry and identified as *P. denitrificans* NasC (Significance score, 139; 49% coverage; and expect value, 3.4 × 10^−10^). Notably, this NasC band was not present when NarJ‐loaded beads were incubated with heat‐treated lysates from anaerobically grown cells using ${\text{NH}}_{4}^{+}$ as N‐source and ${\text{NO}}_{3}^{-}$ as respiratory electron acceptor. Instead, a much larger band at ~ 141 kDa was bound by the NarJ‐loaded beads, which mass spectrometry analysis confirmed as NarG (Significance score, 154; 45% coverage; and expect value, 2.3 × 10^−9^; see Fig. 7, lane 7). In both cases, the staining intensities of NarJ:NasC or NarJ:NarG bands from beads were similar, consistent with formation of 1:1 protein complexes. Finally, assays were performed using lysate from cells grown anaerobically with ${\text{NO}}_{3}^{-}$ as both respiratory electron acceptor and sole N‐source. In this case, NarJ‐loaded beads readily bound both NasC and NarG proteins from this cell lysate (Fig. 7, lane 8). Notably, the NarJ band was around twice the intensity of the NasC and NarG bands, indicating 50% of NarJ being bound in a 1:1 complex with NasC and the remaining 50% bound in a 1:1 complex with NarG. The results therefore imply comparable specificity of NarJ for both types of ${\text{NO}}_{3}^{-}$ reductase under these experimental conditions.

## Discussion

This study has established a key role for the *narJI* genes in assimilatory ${\text{NO}}_{3}^{-}$ reduction in *P. denitrificans*. While NarI, an integral‐membrane quinol‐dehydrogenase, could conceivably play a role in electron transport from quinol to the NADH‐dependent ${\text{NO}}_{3}^{-}$/${\text{NO}}_{2}^{-}$ reductase NasBGC, a recognized electron‐transport pathway is encoded by the *nas* gene cluster (Gates *et al.*, 2011) while a maturation protein for NasC is notably absent. Therefore, this present study reveals NarJ as the assembly factor for NasC. An emerging theme has been biochemical overlap between Nar and Nas components (Gates *et al.*, 2011; Goddard *et al.*, 2017) and this now extends this to functional maturation. There is no apparent REMP in the *P. denitrificans nas* gene cluster and indeed genome analyses fail to detect genetic interactions between NarJ and NasC in any *nas* cluster currently in prokaryotic databases. However, *nas* gene clusters are very diverse and frequently do not contain all of the structural components to assemble the full ${\text{NO}}_{3}^{-}$ assimilation pathway, e.g. transport systems, catalytic and electron transport proteins (Luque‐Almagro *et al.*, 2011). A clear example of this is now illustrated by a REMP in the Nar cluster serving in assembly of the Nas system.

The NarJ family of REMPs includes DmsD and TorD that chaperone the catalytic subunits during maturation of dimethylsulfoxide and trimethylamine N‐oxide reductases, respectively. Previous bioinformatics analyses of the taxonomically diverse NarJ subfamily revealed a close association between each chaperone and a specific complex iron‐sulfur molybdoenzyme respiratory system, or the operon encoding that system (Bay *et al.*, 2015). It was also concluded that NarJ members demonstrated the strictest conservation within the subfamily with respect to target sequence motif conservation and their association with operons encoding the respiratory membrane‐bound ${\text{NO}}_{3}^{-}$ reductase. Here, however, we have presented new insights that extend the specificity of NarJ to functional maturation of the assimilatory ${\text{NO}}_{3}^{-}$ reductase NasC, as well as the respiratory ${\text{NO}}_{3}^{-}$ reductase NarG. Despite both classes of ${\text{NO}}_{3}^{-}$ reductase being cytoplasmic iron‐sulfur and Mo[PGD]_2 _binding catalysts, they are very different sizes and show substantial sequence divergence, exemplified by the NasC and NarG proteins from *P. denitrificans* sharing only ~25% amino acid sequence identity.

**Figure 8:**
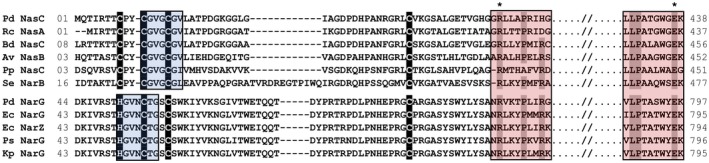
Multiple sequence alignment of representative catalytic subunits for assimilatory and membrane‐bound respiratory ${\text{NO}}_{3}^{-}$ reductases. The organisms selected were: Pd, *Paracoccus denitrificans* Pd1222; Rc, *Rhodobacter capsulatus* E1F1; Bd, *Bradyrhizobium diazoefficiens* USDA 110; Av, *Azotobacter vinelandii* DJ; Pp, *Pseudomonas putida*; Se, *Synechococcus* sp strain PCC 7942; Ec, *Escherichia coli* K‐12; Ps, *Pseudomonas stutzeri*; and Kp, *Klebsiella pneumoniae*. N‐terminal [4Fe‐4S] cluster coordinating residues are highlighted in black. Regions shaded in blue and red show predicted sequence regions required for [4Fe‐4S] (and subsequent Mo[PGD]_2_) loading and a conserved solvent‐exposed salt bridge in Mo/W [PGD]_2_ family proteins (Arias‐Cartin *et al.*, 2016), respectively. Asterisks denote the conserved R58 and E437 residues for *P. denitrificans* NasC.

In *E. coli*, NarJ has been proposed to interact with NarG via the first 50 residues of the partially folded protein, largely by hydrophobic interactions (Li and Turner, 2009). In NarG, these residues precede the N‐terminal FS0 iron‐sulfur cluster binding motif. However, only five amino acids precede the corresponding iron‐sulfur cluster binding motif in NasC, which makes it unlikely that this constitutes the only binding region for NarJ. There must instead be additional features of the extended/partially folded NarG and NasC peptides, beyond the N‐terminal iron‐sulfur cluster binding motif that share sufficient common features to facilitate NarJ binding. Consistent with this outlook, other key features for NarJ‐assisted cofactor insertion in Mo/W‐[PGD]_2_ family members are conserved in NasC (Fig. 8). Firstly, a sequence ^11^CGVGCGV^17^ is present within the N‐terminal/proximal iron‐sulfur cluster binding motif in *P. denitrificans* NasC and other assimilatory ${\text{NO}}_{3}^{-}$ reductases that has similarity to the ^49^H/CGVNCTG^55^ sequence required for FS0 insertion and subsequent Mo[PGD]_2_ loading for *E. coli* NarG. (Lanciano *et al.*, 2007; Arias‐Cartin *et al.*, 2016) Secondly, further residues that may support formation of an arginine‐glutamate salt‐bridge identified by Arias‐Cartin and co‐workers for correct protein‐folding of Mo/W‐[PGD]_2_ family holoenzymes, (Arias‐Cartin *et al.*, 2016) are also conserved in NasC (i.e. R58 and E437). Therefore, there must be sufficient similarity in key peptide regions and chaperone recognition sites beyond the N‐termini of both *P. denitrificans* NarG and NasC to enable functional interactions with NarJ to take place.

Furthermore, in order for a REMP to be utilized for assembly of a partner redox enzyme, the genes encoding both proteins must be expressed under the same growth conditions, such that chaperone availability coincides with biosynthesis of the target. In *E. coli*, *narJ* is expressed under anaerobic growth conditions so that the NarJ protein can mature the anaerobic respiratory ${\text{NO}}_{3}^{-}$ reductase. For other facultative aerobic microorganisms that perform ${\text{NO}}_{3}^{-}$ assimilation, both aerobic and anaerobic expression of *narJ* is essential; such that the chaperone is present to assist maturation of the target holoenzyme regardless of oxygen tension. Here we have shown how a separate mode of gene regulation exists to enable expression of *narJ* during both anaerobic and aerobic ${\text{NO}}_{3}^{-}$ metabolism so that it can perform these dual functions in ${\text{NO}}_{3}^{-}$ assimilation and denitrification. Such regulation may be widespread in bacteria that utilize ${\text{NO}}_{3}^{-}$ for multiple purposes. Consistent with this proposal, bioinformatic analysis reveal widespread co‐conservation of *narJ* (as part of the *narGHJI* cluster) and *nasC* in exemplar bacteria that can both respire and assimilate ${\text{NO}}_{3}^{-}$ (Gates *et al.*, 2011; Luque‐Almagro *et al.*, 2011). That said, one notable exception is *Bradyrhizobium diazoefficiens*, in which the Nap system is responsible for ${\text{NO}}_{3}^{-}$ respiration. Therefore, in some cases there is scope for additional chaperones beyond NarJ in maturation of NasC family proteins in bacteria and this prospect remains to be explored further in the future.

## Experimental procedures

### Bacterial strains, media and growth conditions

All bacterial strains and plasmids used in this study are listed in Table 1. *P. denitrificans* and *E. coli* strains were routinely cultured in Luria‐Broth (LB) medium, at 30 and 37°C, respectively. Where appropriate, antibiotics were added at the following final concentrations: ampicillin (Ap) or carbenicillin (Cb), 100 µg/ml; gentamycin (Gm), 20 µg/ml; and kanamycin (Km), 25 µg/ml. For selection of *P. denitrificans*, rifampicin (Rif), 50 µg/ml or spectinomycin (Sp), 25 µg/ml was used. A defined minimal salts medium was used for growth of *P. denitrificans*, which contained 29 mM of Na_2_HPO_4_, 11 mM of KH_2_PO_4_ and 0.4 mM of MgSO_4_ at pH 7.5. Cultures were supplemented with 30 mM of sodium succinate as carbon source and 2 ml/L of a Vishniac and Santer trace elements solution that was prepared as previously described (Gates *et al.*, 2011), except for omission of sodium molybdate as appropriate. For aerobic batch growth, each inorganic‐N source (either NH_4_Cl, NaNO_3_ or NaNO_2_) was added at 10 mM and 50 ml of culture medium was placed in 250 ml conical flasks and rotated at 180 rpm to ensure maximal aeration of cultures. Anaerobic growth experiments were performed in minimal medium containing 20 mM of NaNO_3_ (or NaNO_2_ in selected experiments) as terminal electron acceptor with 10 mM of NH_4_Cl provided as inorganic‐N source as necessary. For experiments investigating ${\text{NO}}_{3}^{-}$ respiration, 400 ml of culture medium was placed in 500 ml sealed Duran bottles with gas‐tight silicone septa for anaerobic sampling and cells were allowed to consume the initial oxygen and enter anaerobic growth. Batch cultures were inoculated with 1% (v/v) of an overnight minimal medium pre‐culture. Cell growth was monitored by measuring attenuance (*D*) of cultures at 600 nm.

**Table 1:** Bacterial strains and plasmids used in this study.

Name	Relevant characteristics	Source
Bacterial strains		
*P. denitrificans* Pd1222	Wild‐type strain, Rif^R^	de Vries *et al.* (1989)
*P. denitrificans* Pd2401	Unmarked *narJ^‐^* deletion mutant, Rif^R^	Present work
*E. coli* JM101	Used as host for pK18*mobsacB*‐based plasmids	Messing (1979)
Plasmids		
pJET1.2/blunt	Cloning vector, Ap^R^, Cb^R^	Thermo Scientific
pRK2013	Used as mobilizing plasmid in triparental crosses, Km^R^	Figurski and Helinski (1979)
pK18*mobsacB*	Allelic exchange suicide plasmid, sucrose‐sensitive, Km^R^	Schäfer *et al.* (1994)
pLMB509	Broad‐host range expression vector, Gm^R^	Tett *et al.* (2012)
pBJP011	pK18*mobsacB*‐derivative, construct for *narJ* deletion, Km^R^	Present work
pBJP012	Expression construct for native NarJ in *P. denitrificans*, Gm^R^	Present work
pBJP030	Expression construct for recombinant NarJ (with C‐terminal His_6_‐tag) in *P. denitrificans*, Gm^R^	Present work

### Generation of an unmarked *P. denitrificans narJ* mutant

A construct for deletion of *narJ* was produced using the mobilizable suicide vector pK18*mobsacB*, essentially as described previously (Sullivan *et al.*, 2013). Upstream and downstream regions (~0.6 kb each) flanking *narJ* were amplified by the polymerase chain reaction (PCR) from genomic DNA isolated from *P. denitrificans* Pd1222 in separate reactions using the oligonucleotide primer sets P1/P2 and P3/P4, respectively (Table 2). PCR products were cloned into pK18*mobsacB* to form a suicide plasmid in an *E. coli* JM101 donor strain. Correct assembly and DNA fidelity was verified by sequencing. The suicide plasmid was then transferred to *P. denitrificans* Pd1222 from the donor strain via tri‐parental conjugation, using an *E. coli* helper strain that contained plasmid pRK2013 (Gates *et al.*, 2011). Genomic integration of the suicide vector in *P. denitrificans* was assessed by selection of potential trans‐conjugants for Sp and Km resistance on replica LB‐agar plates. Km resistant *P. denitrificans* colonies were selected and grown to stationary phase, in Km deficient LB medium. Resolution of the suicide vector was assessed by further selection where cells were serially diluted and plated onto modified LB‐agar containing 6% (w/v) sucrose, 10 g/L tryptone, 5 g/L yeast extract and 4 g/L NaCl, at pH 7.5. DNA was isolated from sucrose resistant, Km sensitive colonies and screened by PCR to distinguish deletion mutants and WT revertants using primer pairs P5/P6 (Table 2) that amplify regions of the genome external to *narJ*. PCR products obtained were subsequently sequenced to confirm the unmarked deletion of 558 bp (~80%) from the central region of the gene.

**Table 2:** DNA oligonucleotide primers used in this study.

Primer	Sequence (5ʹ → 3ʹ)
Cloning	
P1 (For.)	GAGAATTCAACCTGCGTCGGCCGCATCC
P2 (Rev.)	GATCTAGACTCGGAAGCTTTTCATGCGA
P3 (For.)	GATCTAGAGCCGTCTGGGAAGAGGCGCA
P4 (Rev.)	GACTGCAGGCCGAGATCATGTGGACAAG
P7 (For.)	GACATATGAAAAGCTTCCGAGCCCTTTC
P8 (Rev.)	GACATATGTCATTGCGCCGGGTTGGCGA
P9 (Rev.)	GACATATGTTGCGCCGGGTTGGCGA
Sequencing	
P5 (For.)	GCAGCAGATCGACGAGATGT
P6 (Rev.)	GCCCAAGCCGTCGAATAC
qRT‐PCR	
*narK1* (For.)	TATCCGCCGACCGACTATAC
*narK2* (Rev.)	GACCGGGATATGCTTGAAGA
*narG1* (For.)	GTATGCCCATACCGACCAGT
*narG2* (Rev.)	CCGGATGTTGTAGTCGATCA
*narH1* (For.)	GGAAAAATGCATCCTGTGCT
*narH2* (Rev.)	AAGCATCACGCCCAGATAAC
*narJ1* (For.)	GGCGACCTCTACGATCTTCA
*narJ2* (Rev.)	CGATAGGTCTCCAGCAGGTC
*narI1* (For.)	ATGACCATCCTGGTCTCGAT
*narI2* (Rev.)	ATGCAGCTTGAAGAGCCAAT
*dnaN1* (For.)	CATGTCGTGGTGGTCACCATAC
*dnaN2* (Rev.)	CTCGCGACCATGCATATAGA

### Complementation of the *∆narJ* strain

Amplification of *narJ* from *P. denitrificans* Pd1222 was performed using PCR primer pair P7/P8 and the product was cloned as an NdeI‐NdeI fragment into the taurine inducible and low‐copy expression plasmid pLMB509 (Tett *et al.*, 2012). Sequencing of the multiple cloning site was performed to confirm the correct gene sequence and orientation of the insert. This construct was conjugated into the ∆*narJ* strain and colonies containing the complementation plasmid were selected by screening for Gm resistance. Control experiments confirmed that *P. denitrificans* was unable to grow using taurine as sole N‐source at concentrations below ~ 10 mM (data not shown). Accordingly, expression of *narJ* in‐trans was induced by supplementing minimal growth media with 1 mM taurine to stimulate NarJ production.

### Assimilatory and respiratory nitrate/nitrite‐reductase activity assays

*P. denitrificans* strains were grown to mid‐exponential phase (*D*_600 nm_ ~ 0.5) after which cells were harvested at 5,500 × g for 10 min at 4°C. Soluble cell‐free extracts were prepared and reactions were performed essentially as previously described (Gates *et al.*, 2011). Methyl viologen (MV) and NADH‐dependent ${\text{NO}}_{3}^{-}$ and ${\text{NO}}_{2}^{-}$ reductase activities were measured in 20 mM HEPES, 150 mM NaCl (pH 7.5) containing either 1 mM of MV or 0.1 mM of NADH as required, in anaerobic reactions at 20°C. Microliter volumes of a 10 mM sodium dithionite solution were added to MV‐dependent reactions to generate the reduced MV^1+^ cation radical electron donor. MV‐dependent reactions were followed at 600 nm, while NADH‐dependent reactions were monitored at 340 nm. Addition of either 1 mM of NaNO_3_ or NaNO_2_ was used to initiate individual reactions (Gates *et al.*, 2011).

### Analysis of nitrate consumption

Extracellular nitrate levels in media samples was determined using a Dionex ICS‐900 ion chromatography system fitted with a carbonate eluent anion exchange column (Ion Pac AS22, 2,250 mm, Thermo Scientific), DS5 conductivity detector and AS40 automated sampler. Samples and reagents were prepared with analytical reagent grade water (Fisher Scientific) and filtered through a 0.2 µm syringe filter prior to use. Automated chromatography runs were performed and ${\text{NO}}_{3}^{-}$/${\text{NO}}_{2}^{-}$‐standard curves generated for sample quantitation using the Chromeleon Software Package (ver. 6.8, Dionex Corp. USA) according to the manufacturer's instructions.

### Gene expression analysis by quantitative RT‐PCR

RNA was isolated from *P. denitrificans* cells grown to mid‐exponential phase. Approx. 30 ml of cell culture was incubated with 12 ml of ice‐cold 95% (v/v) ethanol/5% (v/v) phenol solution for 30 min. Cells were pelleted at 4,000 × g for 10 min at 4°C, and RNA was isolated using the SV Total RNA Isolation Kit (Promega), according to the manufacturer’s instructions. RNA integrity was assessed using an Experion Automated Electrophoresis platform (Bio‐Rad) and StdSens chips (Bio‐Rad). Approx. 2 µg of RNA was reverse transcribed to cDNA using SuperScript II reverse transcriptase and random primers (Invitrogen), according to manufacturer’s specifications. Resulting cDNA was diluted 1 in 5 with nuclease‐free water before use in quantitative PCR reactions. Primers pairs (each at 0.4 µM final concentration) from Table 2 were used to amplify each gene of the *narKGHJI* operon individually. Real‐time transcript quantification was performed using a Sensi‐FAST SYBR No‐ROX kit (Bioline) and Bio‐Rad C1000 thermal cycler equipped with a CFX96 real‐time PCR detection system. Standard curves and primer efficiencies were determined using serially diluted genomic DNA stock (100 ng/µl) previously isolated from *P. denitrificans* Pd1222. Relative gene expression was calculated as described previously (Sullivan *et al*., 2013), with expression normalized to that observed for *dnaN*, a constitutively active ‘house‐keeping’ gene that encodes the β‐subunit of DNA polymerase III (see Table 2 for primer sequences).

### Cloning, expression and purification of NarJ

A ~700 bp DNA section containing *narJ* was amplified by PCR using primers P7 and P9 and cloned into the pLMB509 expression vector as an NdeI‐NdeI fragment to generate an expression plasmid for a recombinant variant of NarJ with C‐ terminal His_6_‐tag. Sequencing confirmed correct orientation and fidelity of the coding region and the expression construct was conjugated into *P. denitrificans* Pd1222. Gm resistant colonies containing pBJP030 were selected. Cultures were supplemented with 10 mM of taurine at the point of inoculation to stimulate overexpression of the NarJ‐His_6_ protein and cells were grown overnight at 30°C in 1 L of LB medium in 2.5 L shake‐flasks rotated at 180 rpm. Cells were harvested at 5,000 × g for 20 min at 4°C and washed once with 20 mM of sodium phosphate buffer (pH 7.5). Following re‐suspension in buffer A (20 mM HEPES, 150 mM NaCl and 25 mM imidazole, pH 7.5), the cell mixture was supplemented with cOmplete™ EDTA‐free protease inhibitor cocktail (Sigma) and 1 mg/ml lysozyme (chicken egg white, Sigma), and incubated at 4°C with gentle mixing for 30 min. Cell lysis was achieved following a 10 min period of incubation with 1% (v/v) Triton X‐100 (Sigma) after which the lysate was supplemented with DNase I (bovine pancreas, Sigma) and RNase A (bovine pancreas, Sigma). Following a further 10 min incubation, the lysate became fluid and insoluble cell debris was removed by ultracentrifugation at 40,000 × g for 60 min at 4°C. NarJ‐His_6_ was purified using immobilized metal affinity chromatography (IMAC, HiTrap Chelating HP, GE Healthcare) columns pre‐charged with Ni^2+^ and equilibrated with buffer A, at 4°C. A flow rate of 1 ml/min was applied. Soluble cell extract was loaded onto the column and unbound protein removed by washing with 4 column volumes of buffer A. Bound protein was eluted over 5 column volumes in 1 ml fractions by application of a linear gradient of 25–500 mM imidazole. Fractions containing NarJ‐His_6_ were determined by SDS‐PAGE and Western‐Blot with anti‐His_6_ conjugate. Purified protein was pooled and dialyzed at 4°C overnight against 20 mM HEPES, 150 mM NaCl, pH 7.5.

### Determining NarJ binding targets by pull‐down assay

Interactions between NarJ and cognate partners were investigated by an *in vitro* pull‐down assay using magnetic beads (His Mag Sepharose^®^ Ni, GE Healthcare) pre‐charged with purified NarJ‐His_6_ protein and soluble heat‐shocked (~85°C, 2 min) extracts prepared from *P. denitrificans* Pd1222 cells grown under defined ${\text{NO}}_{3}^{-}$‐utilizing regimes, essentially as described previously (Gates *et al.*, 2011). Beads were pre‐charged with Ni^2+^ and resuspended by vortex before being dispensed into 50 µl aliquots, washed once with 100 µl analytical‐grade water and then with 200 µl buffer A. Each aliquot was incubated with ~1.5 µM purified NarJ‐His_6_ for 10 min and samples were mixed by gentle rocking. Unbound protein was removed from beads with two washes of 200 µl buffer A. Immobilization of partner proteins was achieved by incubation of NarJ‐His_6_‐charged beads with 500 µl of cell extract (prepared in 20 mM HEPES, 150 mM NaCl, pH 7.5) for 40 min during which samples were continuously mixed by gentle rocking. Protein‐bound beads were then thoroughly washed four times with 200 µl of buffer A. Removal of NarJ‐His_6_‐protein complexes from magnetic beads was achieved by addition of 30 µl of 500 mM imidazole, and protein samples were analyzed using 12.5% SDS‐PAGE gels stained with Coomassie Brilliant Blue to visualize bands. Proteins of interest were identified using MALDI‐TOF/TOF mass spectrometry as described previously (Luque‐Almagro *et al.*, 2013).

## Acknowledgements

This work was supported by the Biotechnology and Biological Sciences Research Council (New Investigator Grant BB/M00256X/1 to AJG).

## Conflict of interest

The authors declare that they have no conflicts of interest with the contents of this article.

## Author contributions

BJP, MJS‐L and MJS designed and conducted experiments, and analyzed data. VML‐A, GR, SJF and MDR designed experiments and discussed results. DJR and AJG conceived and coordinated the study, designed experiments, analyzed data and wrote and revised the manuscript in agreement with all authors.
